# Primer on Radiation Oncology Physics, authored by Eric Ford. First edition, CRC Press. 2020. ISBN 978‐1‐138‐59438‐9 (hbk).

**DOI:** 10.1002/acm2.13196

**Published:** 2021-02-17

**Authors:** Minsong Cao

**Affiliations:** ^1^ Department of Radiation Oncology University of California Los Angeles CA USA

## 1 OVERVIEW

As suggested by its title, *Primer on Radiation Oncology Physics* aims to provide a comprehensive introduction to the fundamentals of radiation oncology physics, and has successfully done so with intelligible text and interactive video tutorials. It is intended as a textbook for students and trainees at various levels of training, including medical physics graduate students, clinical radiation oncology and physics residents, and students in medical dosimetry and radiation therapist educational programs. Teaching and learning medical physics are both challenging tasks. While several textbooks are available for medical physics graduate students, such as the classic *Khan's The Physics of Radiation Therapy*, there is a limited selection for students and instructors involved in the training programs of other professionals in radiation oncology with limited physics and mathematics backgrounds. This book fills in this gap and is designed to support clinical residents, medical dosimetrist, and radiation therapist trainees who are taking their first medical physics course. The text presents physics principles and knowledge in basic language with interactive video tutorials for a broad audience. For medical physics trainees, it is not designed to replace existing textbooks of a graduate‐level medical physics course, but it serves well as a great study review resource for graduate students as well as for board exam preparation.

## 2 CONTENTS AND CHAPTERS

This book has over 350 pages and consists of 27 chapters. These chapters are organized and structured in a logical sequence beginning with basic radiation physics principles and progressing to various physics aspects involved in modern clinical radiation oncology. The text starts with an introduction to the basic physics principles, quantities, and radiation interactions as well as radiation production (chapters 1‐9). It is followed by descriptions of the properties of megavoltage photon and electron beams, treatment planning, and radiation measurements. The last section of the book focuses on advanced topics such as quality assurance, image guidance, and specific treatment techniques including SBRT/SRS, TBI, TSET, and particle therapy. This book not only covers an impressively broad range of medical physics topics, but is also up to date with modern advanced technologies in radiotherapy. For example, MR‐guided linac and MR‐guided radiation therapy are discussed in Chapter 9 and 21, respectively, which present the current frontiers in radiation therapy. Instead of providing a comprehensive bibliography, corresponding chapters in the classic textbooks such as Khan and McDermott are listed at the end of each chapter for further in‐depth reading. A set of 10 questions is provided for the readers to enhance their learning and understanding by solving problems related to each chapter. A total of 60 teaching videos are provided free on the publisher's website (https://routledgetextbooks.com/textbooks/9781138591707/interactive‐videos.php). Each video is 10‐15 minutes in length and corresponds to a specific topic in the text. These videos mimic an interactive classroom learning environment with whiteboard drawings and embedded quiz questions that can be invaluable to enhance the learning experience and effectiveness.

## 3 FEATURES AND HIGHLIGHTS

The strength of this book lies in its simplicity. It is written concisely with words that are intelligible to the readers who do not possess strong physics and mathematics backgrounds. The text is supplemented by numerical color illustrations to facilitate the learning of complicated physics concepts and processes. In addition, most pictures related to radiation treatment plans and dose distributions are from commercial treatment planning systems with actual patient images, making it easy to connect the knowledge to clinical practice.

The most exceptional feature of this book is its interactive video series. As an instructor of a radiation physics course for clinical residents in a classroom setting for more than a decade, I found it extremely difficult to teach the course online this past year due to the social distancing requirement caused by the pandemic. To facilitate online classroom interaction and engagement, I started exploring the flipped classroom model by recording the lectures prior to class. However, it was very time consuming and also required extensive video editing skills. As a result, I was only able to flip a small portion of the lectures although they were well received by our residents. I was thrilled to find that this book provides a comprehensive series of teaching videos that can be very helpful in flipping the entire course into a more powerful interactive learning experience.

## 4 SUMMARY

In my opinion, *Primer on Radiation Oncology Physics* by Eric Ford is an excellent textbook for diverse trainees in radiation oncology who take the first course in radiation therapy physics. It is also valuable to medical physics graduate students and residents as a refresher and review resource. The book is ideally suited for new teaching models with interactive videos and extensive problem sets, and it directly meets the educational needs of radiation oncology professionals with diverse backgrounds.

### About the reviewer

Minsong Cao, Ph.D., DABR, FAAPM, is an Associate Professor and Associate Vice Chair of Education of the Department of Radiation Oncology, University of California, Los Angeles.

